# Notes and Notices

**Published:** 1897-07

**Authors:** 


### NOTES AND NOTICES.

Dr. H. P. Holmes, of Omaha, Nebraska, was married at high noon of June 17th, 1897, to Miss Lulu A. Mooney, at the home of the bride, Fremont, Nebraska. The happy couple will take up their residence at Oregon, Illinois, the home of the Doctor in his boyhood, and there the Doctor will resume the practice of medicine. We wish him much joy and happiness.

"The Open Court," June number, has for frontispiece a handsome portrait of Pythagoras, with an article upon his life. Other articles are: "The Department of Police as a Means of Distributing Charity;" "Historical Sketch of the Jews since the Captivity," and "The Immortality of the Anti-Vivisection Movement." Single copies, 10 cents; annually, $1. The Open Court Publishing Company, Chicago.

Governor Stephens, of Missouri, is not afraid to speak out. Not long ago he appointed homeopathic physicians over one of the State's asylums for the insane. The following, from the New York *Sun*, well shows the effect on the allopaths and the plucky Governor's reply to that effect:

### AT WAR WITH THE DOCTORS.

Gov. Stephens and the Allopathic School Seem to be at Swords' Points.

Jefferson City, Mo., May 6th.—Gov. Stephens was asked to-day what he thought of the resolutions adopted by the Linton District Medical Society at Mexico, Mo., on Wednesday, in which he was censured for placing the Fulton Lunatic Asylum under the control of the homeopathic school. He said:

"These resolutions remind me of some adopted by some gentlemen who met, not many years ago, on a noted occasion. They resolved—'first, that this country is for the Lord's people; second, that we are the Lord's people, and therefore the earth and the fullness thereof is ours, and we shall govern ourselves accordingly.'

"The allopathic physicians are not talking about me any more bitterly than they are about themselves, and if they can stand it, I am sure I can. I never saw any two of them in my life agree upon any one proposition, with the exception that they despise the homeopathic school and all those who differ from them. I was elected on a platform which promised equal rights to all and special privileges to none. I will say that, while it is not my intention to make any threats, if the allopathic doctors continue to amuse themselves by passing slanderous resolutions against me because of this action I will be forced to turn over, instead of one institution to the homeopaths, every institution in the State to them within the next two years."

Gov. Stephens will not lack provocation to execute his threat. To-night the Central District Medical Society of Missouri and the Southeast Missouri Medical Association passed caustic resolutions declaring that the Governor had prostituted his high office to base purposes, and characterizing him as a disgrace to the State.

What gorgeous inquisitors these irate doctors would make if they had the power!—*The Homeopathic Envoy.*

"The International Journal of Surgery" will be sent to any subscriber for one year, beginning with the June number, and payment may be made any time before December 31st, 1897. Address International Journal of Surgery Company, 106 and 108 Fulton Street, New York.

E. B. Treat & Co., publishers of so many of the books reviewed in the pages of The Homeopathic Physician, have removed to a new building, 241 and 243 West Twenty-third Street, near Seventh Avenue, New York. Messrs. Wm. H. and Edwin C., sons of Mr. E. B. Treat, are herewith admitted to membership.

